# Discovery of an EP300 Inhibitor using Structure-based Virtual Screening and Bioactivity Evaluation

**DOI:** 10.2174/0113816128298051240529113313

**Published:** 2024-06-03

**Authors:** Dabo Pan, Yaxuan Huang, Dewen Jiang, Yonghao Zhang, Mingkai Wu, Minzhen Han, Xiaojie Jin

**Affiliations:** 1 Department of Medical Technology, Qiandongnan Vocational and Technical College for Nationalities, Kaili 556000, China;; 2 Department of Pharmacy, The Second Affiliated Hospital of Guizhou Medical University, Guizhou Medical University, Kaili 556000, China;; 3 College of Pharmacy, Gansu University of Chinese Medicine, Lanzhou 730000, China

**Keywords:** EP300, inhibitor, structure-based virtual screening, binding free energy calculation, molecular dynamics simulations, neurodegenerative disease

## Abstract

**Background:**

EP300 (E1A binding protein p300) played a significant role in serial diseases such as cancer, neurodegenerative disease. Therefore, it became a significant target.

**Methods:**

Targeting EP300 discovery of a novel drug to alleviate these diseases. In this paper, 17 candidate compounds were obtained using a structure-based virtual screening approach, 4449-0460, with an IC_50_ of 5.89 ± 2.08 uM, which was identified by the EP300 bioactivity test. 4449-0460 consisted of three rings. The middle benzene ring connected the 5-ethylideneimidazolidine-2,4-dione group and the 3-F-Phenylmethoxy group.

**Results:**

Furthermore, the interaction mechanism between 4449-0460 and EP300 was explored by combining molecular dynamics (MD) simulations and binding free energy calculation methods.

**Conclusion:**

The binding free energy of EP300 with 4449-0460 was -10.93 kcal/mol, and mainly came from the nonpolar energy term (ΔG_nonpolar_). Pro1074, Phe1075, Val1079, Leu1084, and Val1138 were the key residues in EP300/4449-0460 binding with more -1 kcal/mol energy contribution. 4449-0460 was a promising inhibitor targeting EP300, which had implications for the development of drugs for EP300-related diseases.

## 1 INTRODUCTION

EP300 is a significant transcriptional cofactor involved in gene expression regulation and cellular signaling. In addition, EP300 played a key role in a variety of cellular processes, including cell proliferation, differentiation, apoptosis, and DNA repair [1-6]. Histone acetyltransferase of EP300 was strongly associated with the progression of bladder cancer [7], breast cancer [8], lung cancer [9], and some kinds of other cancers [10]. Rational modulation of EP300 was a potentially effective way to treat cancer. Therefore, the discovery of inhibitors targeted at EP300 is beneficial in providing new drug candidates for tumor therapy.

CBP (the cyclic-AMP response element binding protein) and EP300 were highly homologous bromodomain-containing transcriptional coactivators that were involved in many cellular pathways relevant to oncology [11-13]. The EP300 protein structure consists of several structural domains, mainly including two transactivation domains and a central chromatin association and modification region. Two transactivation domains consisted of TAZ1/CH1 (transcriptional-adaptor zinc-finger domain 1/cysteine/histidine-rich region 1), KIX (kinase inducible domain of CREB interacting domain), ZZ(ZZ-type zinc finger domain), TAZ2/CH3(transcriptional-adaptor zinc-finger domain 2/cysteine/histidine-rich region 3) and IBiD (IRF3-binding domain). The central chromatin association and modification region included bromodomain, PHD (plant homeodomain finger), and KAT11 (lysine acetyltransferase domain) [14-18].

Specific inhibitors targeted EP300 were now explored and developed like CPI-1612 [19], DS-9300 [20], GNE-781 [21], CBP30 [22], R-2 [23], PF-CBP1 [24], TPO146 [25], UMB298 [14], CPI-637 [26], Y08197 [27], GNE-272 [28] and several PROTACs degraders [29-38]. These inhibitors were widely used both as chemical tools to study the biological role of their targets in living organisms and as candidates for drug development against several cancer variants and human disorders. However, the current study was not yet systematic, and most of the inhibitors were still in the preclinical stage of the study. Furthermore, none of the PROTAC degraders were orally active because they consisted of two small- molecule ligands joined together by a linker and had molecular weights of 800-1200, much higher than traditional oral drug molecules [34]. The discovery of efficient compounds targeted EP300 provided some assistance in the development of new drugs. Currently, integrating computer-aided drug design and activity testing was an effective method for the rapid discovery of candidate compounds [39-53]. In this paper, 4449-0460, a potential targeted EP300 inhibitor was uncovered using structure-based virtual screening and bioactivity evaluation.

## 2 MATERIALS AND METHODS

### 2.1 Structure-based Virtual Screening

The virtual screening was performed using Schrödinger 2017 (Schrödinger, Inc.) [54]. First, the EP300-inhibitor complex structure was retrieved from the Protein Data Bank database (PDB ID code:5NU5) [55], and pre-processed using the Protein Preparation Wizard module, including gas signing partial charges such as His, Asp, and Arg, adding hydrogen atoms, and minimizing the energy with the OPLS-2005 force field [56]. The water formed water bridges with the ligand and Try1089 was retained, and other crystal holographic waters were removed [57, 58]. The centre mass of the inhibitor in the EP300-inhibitor complex was defined as the binding site, and the receptor grid box was set to 20 Å × 20 Å × 20 Å. The small molecules of the commercial libraries Chemdiv and Specs were screened by Lipinski’s Rule and generated possible states at target pH 7.0 ± 2 in the Epik module [59].

**Fig. (1):**
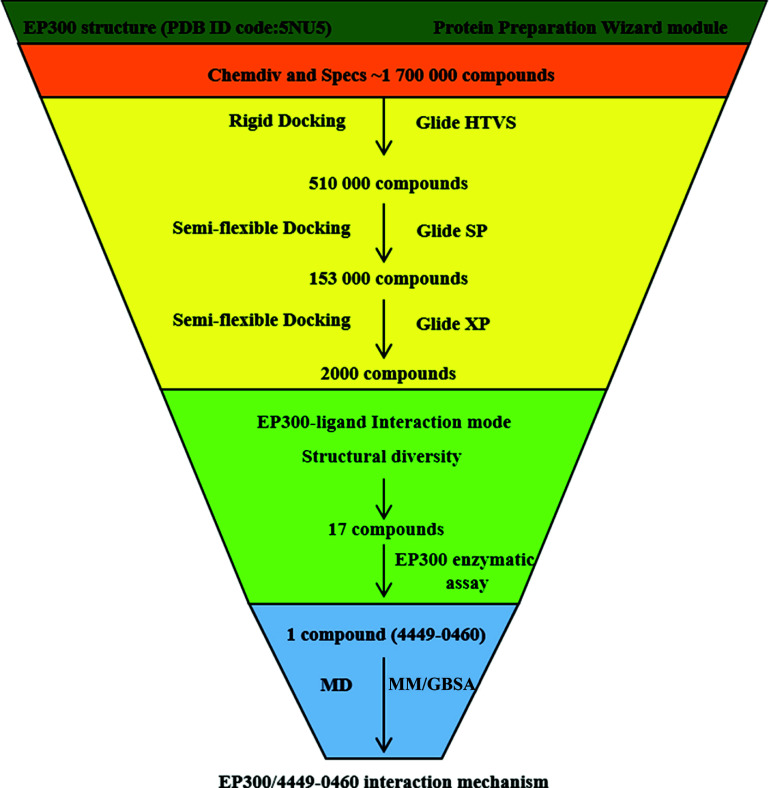
The flowchart of the discovery of an EP300 inhibitor using structure-based virtual screening and bioactivity evaluation.

The Glide high throughput virtual screening (HTVS) scoring mode, the Glide standard precision (SP) scoring mode, and the Glide extra precision (XP) scoring mode in the Virtual Screening Workflow module were used one by one for screening the pre-prepared small molecules (Fig. **1**) [60, 61], for the next step, the top 30% of small molecules scored in each docking step were retained. The top 2000 small molecules in the XP scoring ranking were selected and the binary fingerprints were calculated using the canvas module [62]. One hundred clusters were obtained based on the binary fingerprints properties, and the compounds with the top 2 docking scores in each category were selected. Finally, according to visual inspection, 17 compounds with rational binding modes and good docking scores were chosen and purchased for bioactivity evaluation.

### 2.2 *In vitro* EP300 Enzymatic Assay

The inhibitory activity of compounds that inhibit EP300 was determined by time-resolved fluorescence resonance energy transfer technology by detecting H4 peptide (bH4KAc4 sequence: HSGRGK(Ac)GGK(Ac)GLGK(Ac)GGAK(Ac)RHRK-Biotin-OH) acetylation. The assay was conducted following the instructions of the assay kits. The enzymatic reactions were performed in duplicate at room temperature overnight in a 50 µl mixture containing 50 μM Tris-HCl, pH 7.4, 0.01% Triton X100, 1mM DTT, a test compound, and EP300 enzyme. Luminescence signals were measured using a BioTek Synergy 2 microplate reader. EP300 activity assays were performed in three duplicates at each concentration. The IC_50_ values were calculated from a series of percent inhibition values determined at a range of inhibitor concentrations using Prism software (GraphPad 8.0).

### 2.3 Molecular Dynamics (MD) Simulation and MM/GSA Free Energy Calculation

Gaussian09 was employed to optimize structure and calculate frequency at the HF/6-31G* level. The atomic charges of 4449-0460 were acquired using the restrained electrostatic potential (RESP) method [63]. The parameters of 4449-0460 were generated from the AMBER force field (GAFF) [64]. The standard AMBER force field for bioorganic systems (ff14SB) was employed to describe the protein [65]. Four counter ions Na^+^ were added to neutralize each system. Then, the corresponding systems were solvated using atomistic TIP3P water in a box with at least 10 Å distance around the complex.

AMBER20 was used throughout the whole molecular dynamics simulation for 200 ns [66-69]. Furthermore, to remove bad contacts, the system was minimized by using the steepest descent method switched to conjugate gradient every 2500 steps totally for 5000 steps with a 0.1 kcal/mol Å2 restrains on all atoms. In addition, the system was gently heated from 0 to 310 K over a period of 50 ps by a Langevin thermostat with a coupling coefficient of 0.1/ps. The system was again equilibrated for 500 ps [70]. The binding free energy of 4449-0460 and EP300 was calculated using the MM-GBSA method [71-73], 1000 Snapshots, equally spaced at 200 ps intervals, were extracted from the MD trajectory. For each snapshot, the free energy was calculated for each molecular species (complex, protein, and ligand). The binding free energy was computed as the difference:

*ΔG_bind_* = *G_complex_* – *G_protein_* – *G_ligand_*

The free energy, G, for each species, could be calculated by the following scheme using the MM-GBSA method:

*G* = *E_gas_* – *G_sol_* – *TS*

*E_gas_* = *E*_int_ + *E_ele_* + *E_vdw_*

*E*_int_ = *E_bond_* + *E_angle_* + *E_torsion_*

*G_sol_* = *G_GB_* + *G_nonpolar_*

*G_nonpolar_* = *γSAS*

Here, *E_gas_* was the gas-phase energy; *E_int_* was the internal energy; *E_bond_, E_angle_,* and *E_torsion_* are the bond, angle, and torsion energies, respectively; *E_ele_* and *E_vdw_* were the Coulomb and van der Waals energies, respectively. *E_gas_* was calculated using the AMBER molecular mechanics force field. *G_sol_* was the solvation-free energy and was decomposed into polar and nonpolar contributions. *G_GB_* was the polar solvation contribution calculated by solving the GB equation. The dielectric constant of the solvent was set to 80. *G_nonpolar_* was the nonpolar solvation contribution and was estimated by the solvent accessible surface area (SAS) determined using a water probe radius of 1.4 Å. The surface tension constant γ was set to 0.0072 kcal/mol/Å2. T and S are the temperature and the total solute entropy, respectively. Vibrational entropy contributions were estimated by normal mode analysis. Fifty snapshots were used in the normal mode analysis due to the large amount of computation. Furthermore, to obtain the contribution of each residue to the binding energy, MM-GBSA was used to decompose the interaction energies of each residue involved in the interaction by only considering molecular mechanics and solvation energies without the contribution of entropies.

## 3 RESULTS AND DISCUSSION

### 3.1 The Candidate Compounds by Virtual Screening

Seventeen compounds with high affinity for EP300 were screened based on 2 criterias (Table **1** and Fig. **S1**). 1) The water bridges between compound, water, and Try1089 of EP300 must be detected. 2) High affinity with EP300 theoretically, which was a low docking score. The 2D structure of 17 candidate compounds are shown in Fig. (**S1**).

### 3.2 4449-0460 Inhibits EP300 Enzymatic Activity

**Fig. (2):**
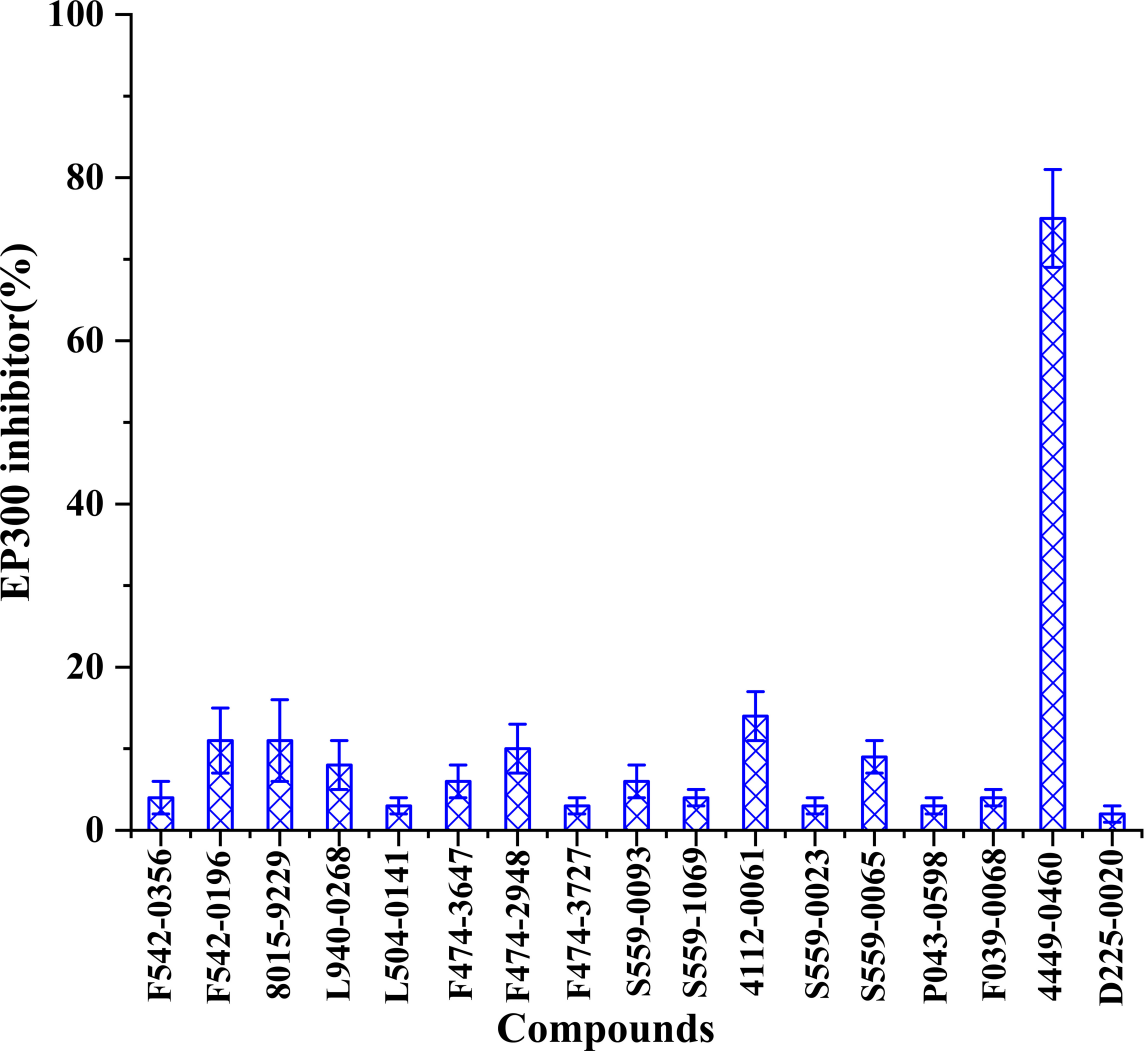
The EP300 inhibitor activity of candidate compounds at a concentration of 10 μM.

**Fig. (3):**
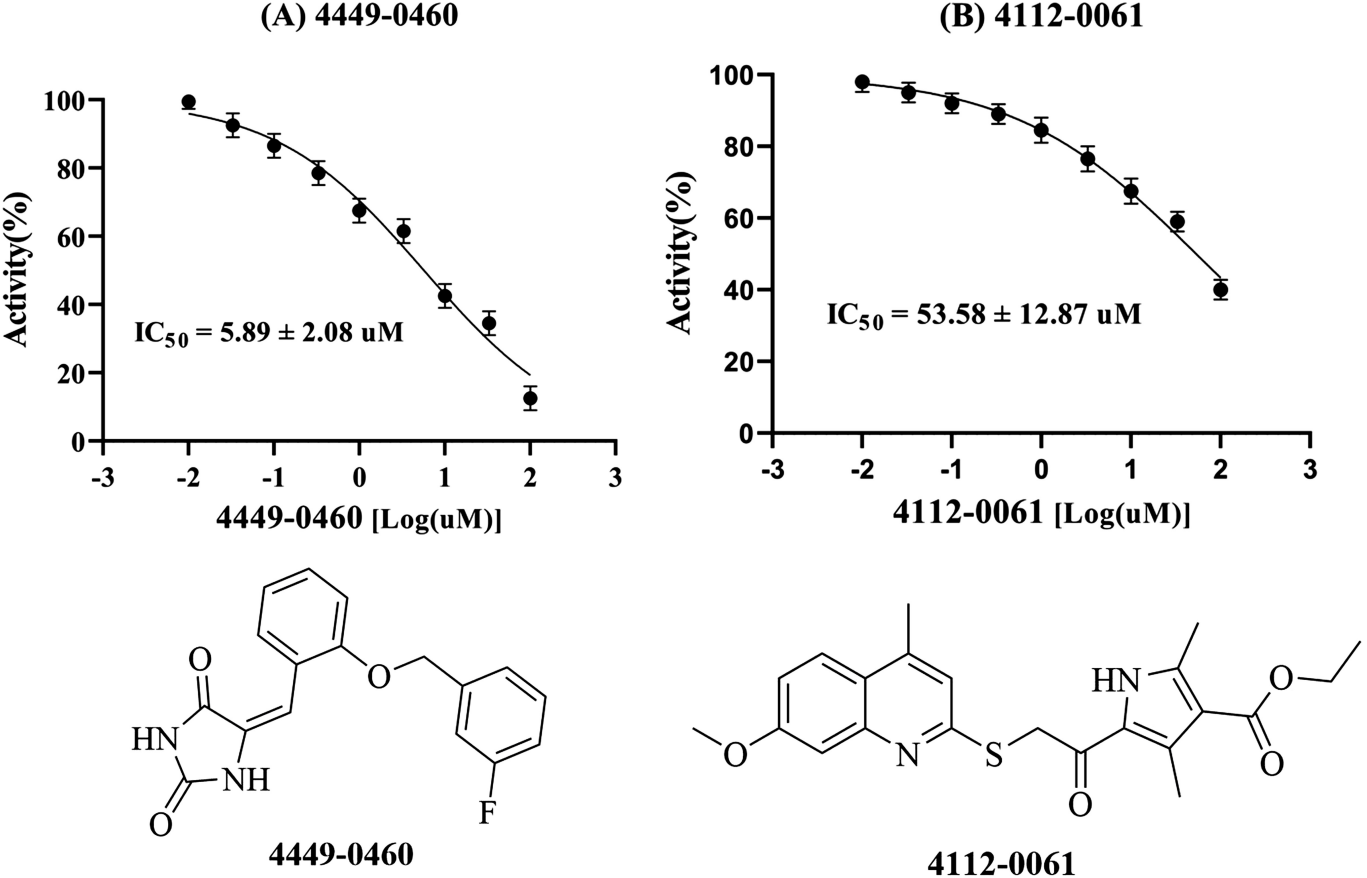
The IC_50_ values of candidate compounds for EP300 inhibition, (**A**) 4449-0460, (**B**) 4112-0061.

Seventeen candidate compounds for inhibition of EP300 enzymatic activity at a concentration of 10 μM were tested (Fig. **2**). Fig. (**2**) shows that 4449-0460 and 4112-0061 demonstrated relatively strong inhibitory activity against EP300, while other candidate compounds illustrated very weak activity. Furthermore, 4449-0460 and 4112-0061 were assessed to suppress the IC_50_ value of the EP300 (Fig. **3**).

The 2D structure of 4449-0460 is shown in Fig. (**S1** and **3**). *In vitro*, a kinase assay was carried out to investigate the influence of 4449-0460 on the kinase activity of EP300. As shown in Fig. (**2**), 4449-0460 inhibited EP300 kinase activity in a dose-dependent manner. The corresponding IC_50_ value was 5.89 ± 20.8 µM (Fig. **3A**), suggesting that 4449-0460 directly inhibited the kinase function of EP300. Compared to 4449-0460, 4112-0061 was less capable of inhibiting EP300, with an IC_50_ of 53.58 ± 12.87 μM (Fig. **3B**).

### 3.3 Interaction Mechanism between 4449-0460 and EP300

**Fig. (4):**
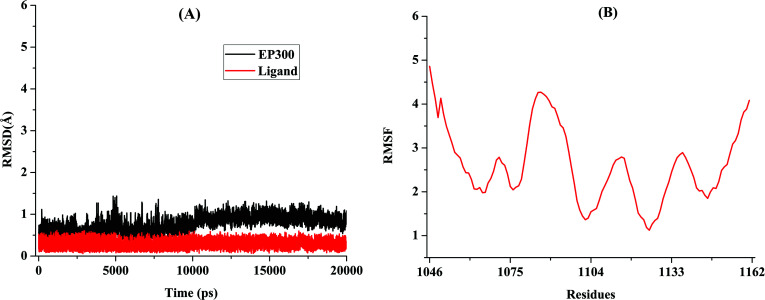
(**A**) Time evolution of RMSD of Cα atoms for the residues around 8Å of 4449-0460 and the heavy atoms of 4449-0460; (**B**) The RMSFs relative to the initial structure for backbone atoms of the EP300.

Two hundred ns molecular dynamics simulation, binding free energy, and amino acid energy decomposition were employed to explore the detailed interaction mechanism between 4449-0460 and EP300. In order to assess the stability of the EP300/4449-0460 complex, we monitored the root-mean-square deviations (RMSD) values of the Cα atoms of 8 Å amino acids around the active site and the heavy atoms of 4449-0460 (Fig. **4A**) [60, 61]. The RMSDs of the Cα atoms of 8 Å amino acids around the active site tended to equilibrate in all simulation times and fluctuated between 0Å and 1.5Å (Fig. **3**). Related to the RMSDs of the Cα atoms of 8 Å amino acids around the active site, The RMSDs of the heavy atoms of 4449-0460 were relatively higher, fluctuating between 0Å and 0.5Å (Fig. **4A**). Furthermore, the RMSF of EP300 is relative to the initial structure of the backbone atoms of the EP300. Amino acid fluctuations were low in all regions except the loop region of EP300 (Fig. **4B**). This suggested that the system reached equilibrium during the simulation. One thousand structures were extracted from 200ns simulation trajectories for the calculation of binding free energy.

**Fig. (5):**
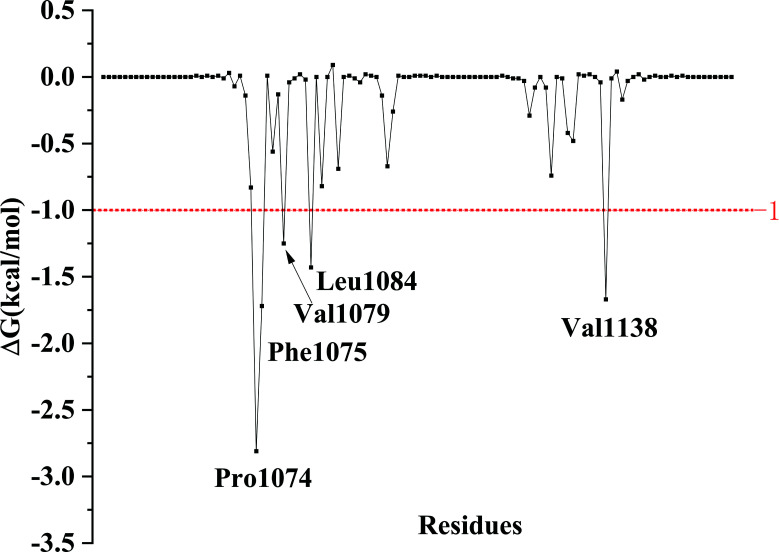
The decomposition energy of amino acids.

The calculated binding-free energy of EP300/4449-0460 was -10.93 kcal/mol (Table **2**). Among the various energy terms, the nonpolar energy term (ΔG_nonpolar_) played a major role in EP300/4449-0460 binding, was -39.54 kcal/mol, while the polar energy term (ΔGpolar) was a positive value (13.19 kcal/mol), which indicated ΔGpolar was unfavorable to bind between EP300 and 4449-0460. In order to explore the key amino acid residues in 4449-0460 binding with EP300, the binding free energy was decomposed into every residue (Fig. **5**). There were five key residues, including Pro1074, Phe1075, Val1079, Leu1084, and Val1138, with an energy contribution of more than -1 kcal/mol.

Furthermore, the average structure of 200 ns MD trajectory was extracted and shown in Fig. (**6**). We extracted the EP300/4449-0460 structures at 0, 40, 80, 120, 160, and 200 ns. The RMSDs of 0, 40, 80, 120, 160, and 200 ns with reference average structure were 0.890, 0.998, 0.892, 1.012, 0.979 and 0.834, respectively. This suggested that the system was stable in all MD simulations and was able to better represent the structure of this simulation process (Fig. **S2**). From Fig. (**6**), the water bridges between the imidazolidine-2,4-dione group of 4449-0460 and Try1089 were detected. Interestingly, the water also formed water bridges with Asn1127. Moreover, 4449-0460 formed two hydrogen bonds with Asn1132 and Phe1074. There was a strong nonpolar interaction between the phenyl motif of 4449-0460 and Val1138. These interactions led 4449-0460 to be better inserted in active pockets, with a relatively rational geometric match and charge match to EP300.

In order to reveal the hydrogen bonds of EP300/4449-0460 interaction, we examined the hydrogen bonds between them, as shown in Table **3**. The main chain of Pro1074 formed a strong hydrogen bond with 4449-0460 with 54% occupation. Furthermore, the hydrogen bond between Asn1132 and 4449-0460 was detected with 58% occupation. The water bridges in 4449-0460, water, Asn1127, and Tyr1089 were relatively stable.

Related to EP300/UMB298 [14], EP300/CPI-637 [26], EP300/Y08197 [27], EP300/GNE-272 (PDB code 5KTX) [28] complex, and EP300/4449-0460,the hydrophilic group of all ligands was inserted into the bottom of the active pocket of EP300 which formed several strong salt bridges with water, Asn1127 and Tyr1089. Moreover, the key hydrogen bond between the ligand and EP300 was left. The ADMET features of 4449-0460 was assessed using ADMETlab3.0 (https://admetlab3.scbdd.com/) [74-76], its Caco-2 permeability, VDss, CLplasma, and Genotoxic Carcinogenicity Mutagenicity Rule were -5.05 (higher than -5.15), 0.653 L/kg (0.04-20 L/kg), 3.255 ml/min/kg (< 5 ml/min/kg low clearance) and 0, respectively. Furthermore, there is a good chance 4449-0460 was not a substrate or inhibitor of CYP2C19, CYP2C9, CYP2D6, and CYP2B6. This indicated that 4449-0460 was a desirable drug candidate with developmental promise.

## CONCLUSION

In this work, we used a virtual screening method based on the EP300 structure to screen large-scale commercial Chemdiv and Specs databases, and 4449-0460 targeted EP300 was identified through biological activity assays. The results showed that 4449-0460 could significantly inhibit the activity of EP300. MD simulation was used to study the binding mechanism of 4449-0460 with EP300, and 449-0460 was well embedded in the active pocket of EP300 due to strong hydrogen bonding and water bridge interactions.

## Figures and Tables

**Fig. (6):**
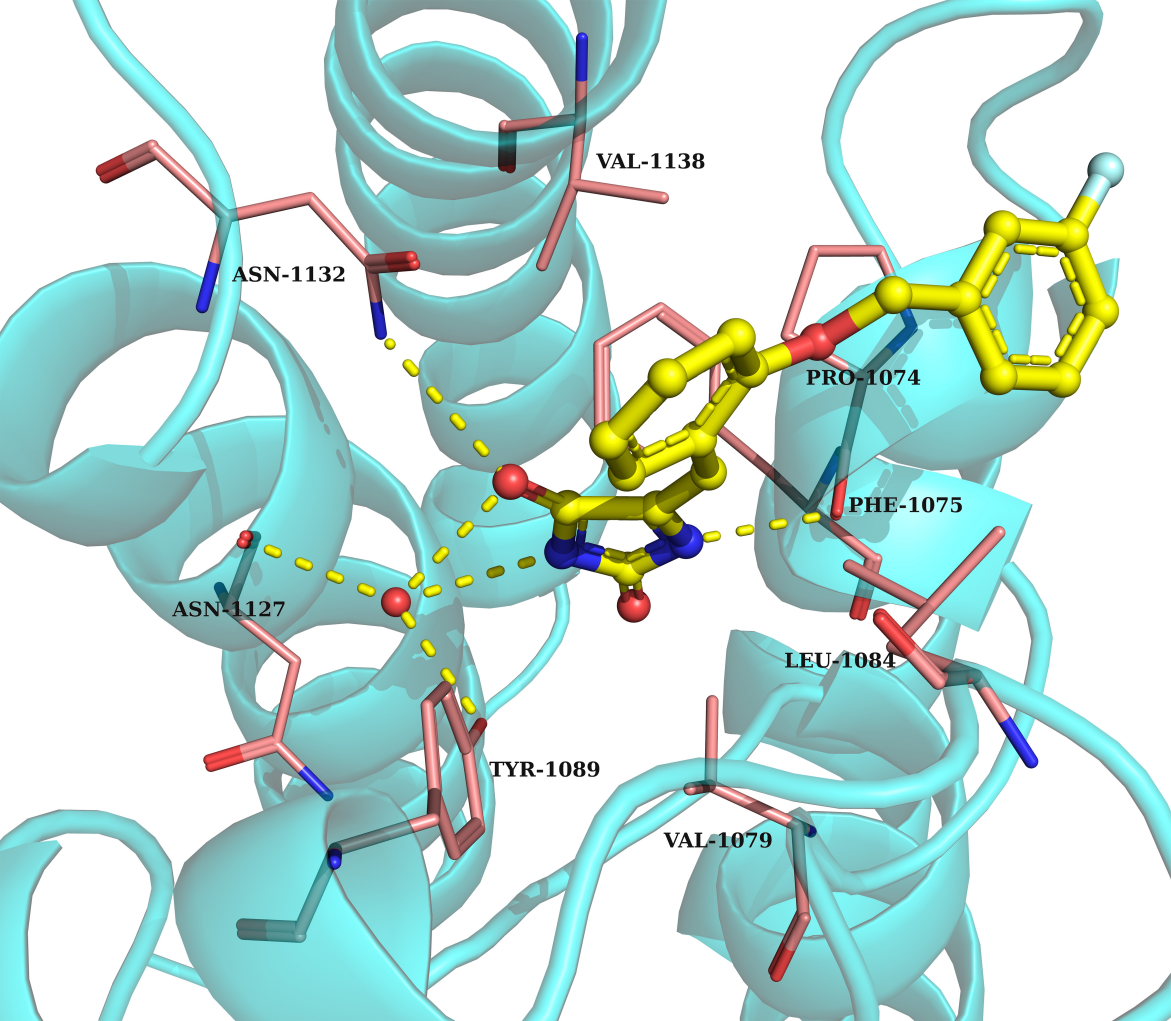
The interactions between EP300 and 4449-0460.

**Table 1:** The inhibitor activity of candidate compounds by virtual screening.

**S. No.**	**Name**	**Docking Score (kcal/mol)**	**Cluster**	**MW**	**10 μM Inhibition %**
1	F542-0356	-8.881	2	392.4079	4
2	F542-0196	-8.544	2	298.2967	11
3	8015-9229	-7.764	6	364.2754	11
4	L940-0268	-7.627	6	406.8236	8
5	L504-0141	-8.471	7	412.4224	3
6	F474-3647	-8.433	10	362.8306	6
7	F474-2948	-8.420	10	425.908	10
8	F474-3727	-7.999	10	394.3197	3
9	S559-0093	-8.211	11	369.4343	6
10	S559-1069	-7.934	11	445.5302	4
11	4112-0061	-7.995	12	412.502	14
12	S559-0023	-9.232	14	333.3806	3
13	S559-0065	-8.463	14	345.4161	9
14	P043-0598	-7.841	15	345.3499	3
15	F039-0068	-8.633	17	282.203	4
16	4449-0460	-7.993	18	312.2951	75
17	D225-0020	-8.423	20	323.3211	2

**Table 2:** The calculated binding free energy (kcal/mol).

**-**	**ΔE_ele_**	**ΔE_vdw_**	**ΔG_sol-np_**	**ΔG_sol-ele_**	**ΔG_nonpolar_^b^**	**ΔG_polar_^a^**	**ΔH_bind_**	**T∆S**	**ΔG_bind_**
4449-0460	-13.28	-34.37	-5.17	26.47	-39.54	13.19	-26.35	-15.42	-10.93

**Note:** ^a^ΔG_polar_ = ΔE_ele_ + ΔG_sol-ele_; ^b^Δ G_nonpolar_ = ΔE_vdw_ + ΔG_sol-np_.

**Table 3:** Percentage occupation of hydrogen bonds between EP300 and 4449-0460.

**Acceptor**	**DonorH**	**Donor**	**Frames**	**Present (%)**	**Average Distance**	**Average Angle**
Pro1074@O	MOL@H2	MOL@N2	10706	54%	2.82	154.19
MOL@O2	Asn1132@HD21	Asn1132@ND2	7252	36%	2.86	154.12
MOL@O2	Asn1132@HD22	Asn1132@ND2	4493	22%	2.85	150.90
MOL@O1	WAT@H1	WAT@O	3044	15%	2.75	159.00
MOL@O1	WAT@H2	WAT@O	5025	25%	2.79	154.34
WAT@O	MOL@H1	MOL@N1	1134	6%	2.82	153.24
Tyr1089@OH	WAT@H1	WAT@O	5110	26%	2.83	157.25
Tyr1089@OH	WAT@H2	WAT@O	2104	11%	2.83	157.99
Asn1127@O	WAT@H1	WAT@O	1005	5%	2.79	152.87
Asn1127@O	WAT@H2	WAT@O	1121	6%	2.90	156.84

## Data Availability

The data and supportive information are available within the article.

## LIST OF ABBREVIATIONS

MD: Molecular Dynamics
RESP: Restrained Electrostatic Potential
RMSD: Root-mean-square Deviations
SP: Standard Precision
