# Supplementary material for: Discovery of an EP300 Inhibitor using Structure-based Virtual Screening and Bioactivity Evaluation
Source: Curr Pharm Des. 2024 Jun 3;30(25):1985–94. doi: 10.2174/0113816128298051240529113313 (PMC11348464; doi:10.2174/0113816128298051240529113313)
Supplement: Supplementary file 1 [file CPD-30-1985_SD1.pdf]

Supplementary Material

Discovery of an EP300 Inhibitor using Structure-based Virtual Screening and Bioactivity Evaluation

Dabo Pan[1,2,*], Yaxuan Huang[1], Dewen Jiang[1], Yonghao Zhang[1], Mingkai Wu[1], Minzhen Han[2,*] and Xiaojie Jin[3,*]

[1]*Department of Medical Technology, Qiandongnan Vocational and Technical College for Nationalities, Kaili 556000, China;* [2]*Department of Pharmacy, the Second Affiliated Hospital of Guizhou Medical University, Guizhou Medical University, Kaili 556000, China;* [3]*College of Pharmacy, Gansu University of Chinese Medicine, Lanzhou 730000, China*

1873-4286/24 $65.00+.00 © 2024 Bentham Science Publishers

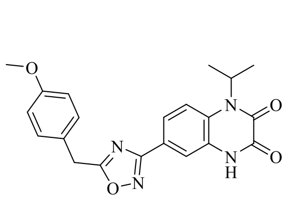

F542-0356

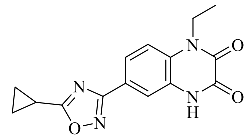

F542-0196

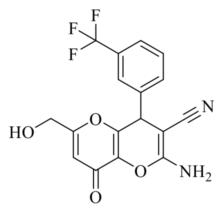

8015-9229

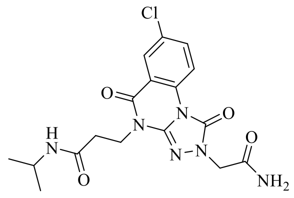

L940-0268

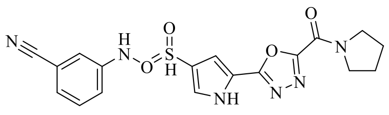

L504-0141

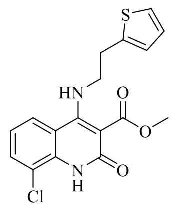

F474-3647

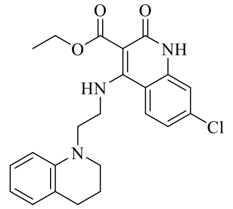

F474-2948

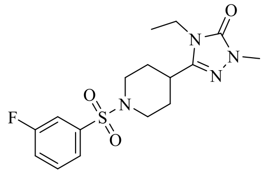

S559-0093

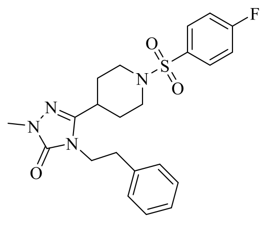

S559-1069

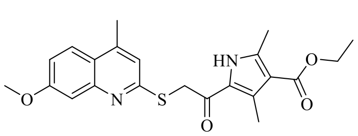

4112-0061

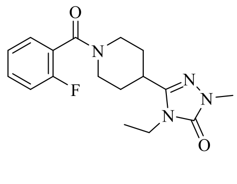

S559-0023

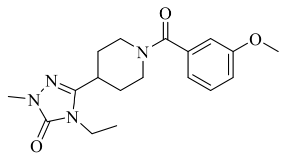

S559-0065

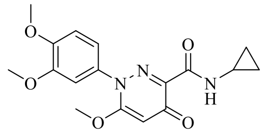

P043-0598

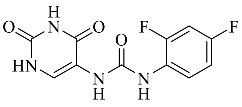

F039-0068

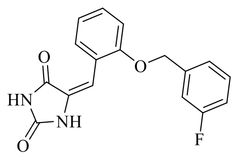

4449-0460

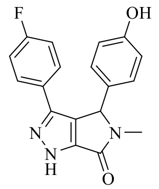

D225-0020

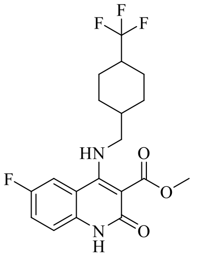

F474-3727

Fig. (S1). The 2D structures of 17 candidate compounds.

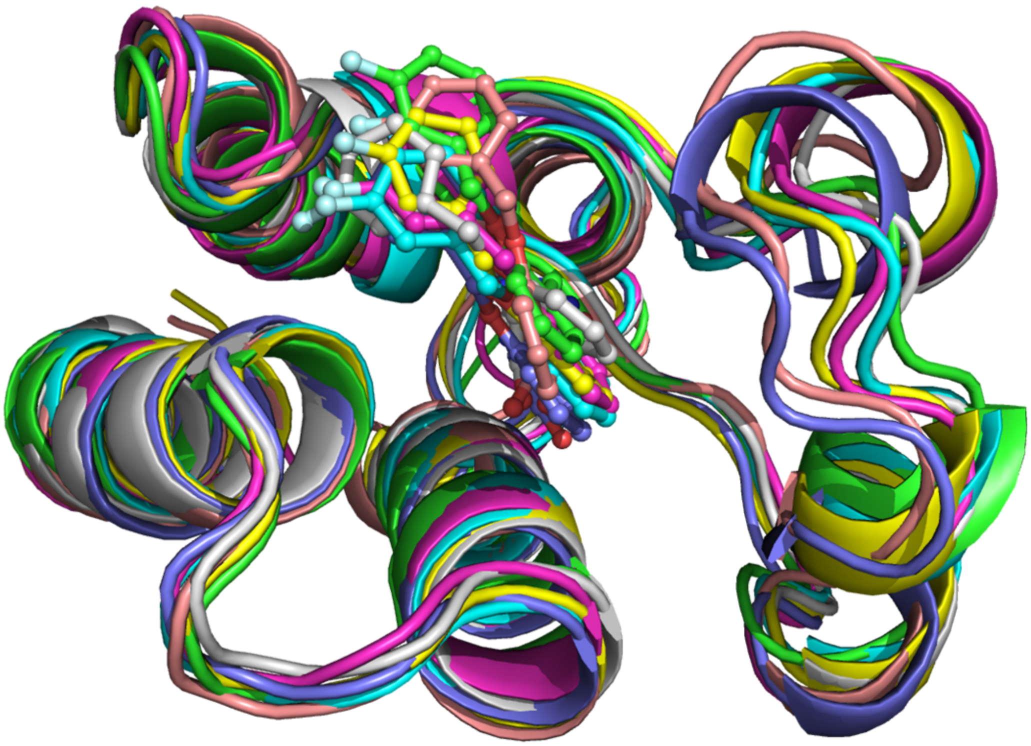

Fig. (S2). Stacked plots of EP300/4449-0460 structures for 0, 40, 80, 120, 160, and 200 ns simulation time (average: green; 0 ns: cyan; 40 ns: magenta; 80 ns: yellow; 120 ns: wheat; 160 ns: white; 200 ns: blue).
